# Rotation of Cotton (*Gossypium hirsutum*) Cultivars and Fallow on Yield and *Rotylenchulus reniformis*

**DOI:** 10.2478/jofnem-2023-0024

**Published:** 2023-06-06

**Authors:** Casiani Soto-Ramos, Terry A. Wheeler, Jonathan Shockey, Cecilia Monclova-Santana

**Affiliations:** Texas A&M AgriLife Extension Service, Lubbock, TX 79403; Texas A&M AgriLife Research, Lubbock, TX 79403; Texas A&M AgriLife Extension Service, Lubbock, TX 79403, currently at United States Department of Agriculture, 1400 Independence Ave SW, Washington, DC 22314

**Keywords:** cotton, *Gossypium hirsutum*, management, reniform nematode, resistance, *Rotylenchulus reniformis*

## Abstract

A three-year rotation of cotton (*Gossypium hirsutum*) cultivars either resistant (R) or susceptible (S) to *Rotylenchulus reniformis* and fallow (F) was examined for effect on cotton yield and nematode density. In year 1, 2, and 3, the resistant cultivar (DP 2143NR B3XF) yielded 78, 77, and 113% higher than the susceptible cultivar (DP 2044 B3XF). Fallow in year 1 followed by S in year 2 (F1S2) improved yield in year 2 by 24% compared with S1S2, but not as much as R1S2 (41% yield increase over S1S2). One year of fallow followed by R (F1R2) had lower yield in year 2 (11% reduction) than R1R2. The highest yield after three years of these rotations occurred with R1R2R3, followed by R1S2R3 (17% less yield) and F1F2S3 (35% less yield). *Rotylenchulus reniformis* density in soil averaged 57, 65, and 70% lower (year 1, 2, 3, respectively) in R1R2R3 compared with S1S2S3. In years 1 and 2, LOG_10_ transformed nematode density (LREN) was lower in F1, and F1F2, than for all other combinations. In year 3, the lowest LREN were associated with R1R2R3, F1S2F3, and F1F2S3. The highest LREN were associated with F1R2S3, F1S2S3, S1S2S3, R1R2S3, and R1S2S3. The combination of higher yield and lower nematode density will be a strong incentive for producers to use the *R. reniformis* resistant cultivars continuously.

The reniform nematode *Rotylenchulus reniformis* causes substantial (>40%) yield losses in cotton (Robinson, 2007; Dyer et al, 2020). Effective management for many years was limited to crop rotation with nonhosts like sorghum or corn (Davis et al., 2003; Robinson, 2007; Stetina et al., 2007). There are several nematicide options available in cotton that have demonstrated effectiveness against *R. reniformis*, including fumigation with 1,3-dichloropropene (Koenning et al., 2007), aldicarb (Lawrence et al., 1990), aldicarb plus oxamyl (Lawrence and McLean, 2000), and fluopyram (Dyer et al., 2020). However, for various reasons (lack of effectiveness under dry conditions, cost, difficulty in application, health and environmental concerns), nematicides have not been a satisfactory management option for all cotton producers. Historically, there were no commercial cotton cultivars with resistance to *R. reniformis* (Robinson, 2007). Recently, *R. reniformis* resistance has been transferred to commercially available cotton cultivars. In 2020, Phytogen released the first *R. reniformis* and *Meloidogyne incognita* resistant cotton varieties (PHY 332 W3FE, plant variety protection [PVP] #202000220; and PHY 443 W3FE, PVP #202000221). In 2021, Deltapine released their first *R. reniformis* and *M. incognita* resistant cotton varieties (DP 2141NR B3XF and DP 2143NR B3XF).

Rotation of cotton with fallow has not been traditionally recommended for *R. reniformis* control compared with rotation with nonhost crops (Robinson, 2007). Prior to the availability of reniform nematode resistant cotton varieties, crop rotation for one or two years in a nonhost like sorghum or corn, followed by cotton was the recommended practice. However, the decision of using deficit-limited irrigation on a grain crop (sorghum or corn) versus leaving land fallow is now more difficult in the Southern High Plains of Texas, due to diminished irrigation availability. In deficit-irrigated cotton production as in the Southern High Plains of Texas, the ability to irrigate entire center pivots has declined with the reduced availability of irrigation water (Mitchell-McCallister et al., 2021). Future scenarios project utilizing only one fourth of the area of a center pivot for irrigated cotton, with transition to increased dryland (rainfed) cotton production. However, for fields infested with *R. reniformis*, it is more likely that part of the land under a center pivot will be left fallow, rather than growing dryland cotton and maintaining higher densities of the *R. reniformis*.

There has been a need previously to incorporate cotton rotation with a nonhost to reduce *R. reniformis* density, thus allowing maximum cotton yields in the subsequent crop. However, rotation with nonhost plants has not always shown consistent yield improvements in *R. reniformis* fields. Field fumigation trials were conducted in two *R. reniformis* infested fields, with one planted continuously to cotton, and the other in a cotton-sorghum rotation (Thames and Heald, 1974). In one year, there was a significant increase in cotton yield with fumigation, where the best fumigation treatment yielded 57% higher than the untreated check in continuous cotton, but no significant yield differences in the field with cotton following sorghum. However, the next year, the reverse was true, with significant yield differences for all chemical treatments when cotton followed sorghum (maximum of 40% yield increase), compared to no significant differences between treatments in the continuous cotton field. Davis et al. (2003) found that in a *R. reniformis* field, continuous cotton yielded consistently less than cotton following corn or following *R. reniformis* resistant soybean. Cotton yield only increased in a *R. reniformis* test after two consecutive years of corn, followed by cotton compared to continuous cotton (Stetina et al., 2007). A corn-cotton-corn-cotton rotation did not significantly increase lint yields.

The reduction of *R. reniformis* density is not always enhanced with planting nonhosts for *R. reniformis* compared with fallow. *Rotylenchulus reniformis* density under fallow maintained similar levels as the nonhost plants (Sunn hemp, Rhodes grass, and Pangola grass) when monitored for 342 days (Caswell et al., 1991). *Rotylenchulus reniformis* density only declined by 52% after 342 days in fallow field plots. Part of the concern over using fallow as opposed to nonhost plants, is that a fallow field would not be irrigated, and *R. reniformis* can survive dry conditions better than many other plant parasitic nematode species. The second, third, and fourth molts occur quickly under moisture stress for *R. reniformis*, and the cuticles of the previous stages form superimposed sheaths which are helpful in reducing water loss (Gaur and Perry, 1991). This nematode may also have coiling of their body, which would reduce the surface area exposed, and thus reduce water loss. Womersley and Ching (1989) found that slow drying like what would be found in agricultural soils, particularly in deeper soil, allowed coiled *R. reniformis* juveniles to survive in greater numbers. Rapid drying and drying down to lower relative humidity (<60%), resulted in higher mortality of *R. reniformis*.

*Rotylenchulus reniformis* has a wide host range, including many weeds (Lawrence et al., 2008; Molin and Stetina, 2016). Therefore, management of weeds (weed-free fallow), would also be important when utilizing fallow as a management tool to reduce the nematode density for a future cotton crop.

Management of *R. reniformis* with either fallow conditions and/or resistant cotton cultivars are important strategies that should be tested. The objective of this study was to test three-year rotations that included various combinations of fallow, reniform nematode resistant and susceptible cultivars, with regards to late summer nematode density and cotton yield.

## Materials and Methods

A *R. reniformis* infested field located at the Texas A&M AgriLife Research and Extension Center, Lubbock, Texas, United States, that had no prior history of use of reniform nematode resistant cultivars was used for the experiment. The soil in the field is an Acuff sandy clay loam (sand = 45%, silt = 26%, clay = 29%), pH = 7.7, organic matter = 0.6%, and CEC=13.2. The plots were 48.8 m long, 4-rows wide, on 1 m centers. The *R. reniformis* resistant cultivar (R) was DP 2143NR B3XF and the susceptible (S) cultivar was DP 2044 B3XF. A third treatment was weed-free fallow (F). The nine different combinations of rotation treatments tested were: R1R2R3 (first letter was for 2020 variety type followed by 1, second letter for 2021 variety type followed by 2, and third letter for 2022 variety type followed by 3), S1S2S3, F1F2S3, F1S2F3, F1R2S3, F1S2S3, R1R2S3, R1S2R3, and R1S2S3. The nine treatments were arranged in a semi-randomized complete block design, with four replications per combination. Each replication was laid across 36 rows (east/west direction, nine treatments x four row plots), and then the four replications were stacked (1.5 m alleys between replications) down the rows (north/south). In 2020 (first year of the trial), the fallow treatments were all placed into rows 1–4, 13–16, 25–28, and rows 33–36. The specific fallow rotation treatment was randomized across the four replications, within those 16 rows. This was done so that the fallow rows were not irrigated in 2020. The cotton was furrow irrigated, typically a week before planting, and then approximately once a month during June, July, and August, unless rainfall negated the need for irrigation. After 2020, fallow treatments (F1F2S3 in 2021 and F1S2F3 in 2022) were irrigated since there were cotton treatments also in the same rows. Cotton was planted on 20 May, 5 June, and 23 May in 2020, 2021, and 2022, respectively. Cotton was harvested on 19, 18, and 16 November in 2020, 2021, and 2022, respectively. Weed control was primarily managed with herbicides. Preplant herbicide treatment was with trifluralin (840 g ai/ha) incorporated, followed by fluometuron applied after planting (preemergence) at 1.12 kg ai/ha. Herbicide applications with glyphosate (630 kg ai.ha) combined with glufosinate (594 g ai/ha) were made to the cotton varieties and fallow areas as needed. S-metolachlor (1.07 kg ai/ha) was also applied prior to the 5^th^ leaf stage to the field in-season (after cotton emergence) to prolong soil herbicide protection. Hand hoeing was also done to handle weed escapes.

Composite soil samples were taken for nematode assays in each plot on 25 April and 17 August in 2020, 14 September in 2021, and 8 September in 2022. Sampling was always conducted either after a significant rainfall event or approximately one week after furrow irrigation, when soil moisture was adequate for sampling. Samples consisted of ten subsamples per plot collected with a narrow-bladed shovel (40 cm depth, 15 cm width at top and 8 cm width at the bottom) to a depth of 20 cm, close to the taproot. The top 6 cm of soil was discarded and then soil from 6–20 cm depth, including some roots was removed. The soil was mixed in a bucket and then a subsample of 750 cm^3^ soil was placed in a plastic bag. The soil samples were refrigerated for <2 weeks before being assayed for plant parasitic nematodes. A pie-pan assay with 200 cm^3^ soil + root fragments was used to extract *R. reniformis* over 48 hours (Thistlethwayte, 1970). The circular pie-pans are made of glass with 18 cm diameter at the base, 22 cm at the top, and 3 cm tall. Three washers were placed in the base of the pie-pan and wire mesh (0.64 cm diameter) laid on top. Two pieces of Kleenex paper tissues (2-ply) were laid on top of the mesh and then the soil sample was placed on the paper tissues. Tap water (250 ml) was gently added to the pie-pan without disturbing the soil, and then the wet paper tissues were arranged around the soil to keep the soil from floating into the water. A cover was placed over the pie-pan to eliminate evaporation. The extracted nematodes were assessed by concentrating the pie-pan water to 100 ml and then counting a 5 ml aliquot. This assay is effective only on live and mobile nematodes.

Cotton was harvested with a modified four-row cotton stripper (John Deere 484), which has a fabricated cage within the body of the harvester that is set on load cells to weigh the harvested cotton. Cotton strippers remove lint, seed, burrs, and other plant debris. Samples were taken from the harvested cotton and ginned to obtain the percentage of the harvest weight that was lint, and lint yield (kg/ha) was calculated. The lint from ginned samples was sent to the Fiber and BioPolymer Research Institute at Texas Tech University, for High Volume Instrument (HVI) testing. The properties measured included micronaire (a relative measure of fiber mass per unit length determined by air permeability), fiber length, strength (force require to break a fiber sample), elongation (the amount that a fiber sample will stretch prior to breakage), uniformity (the ratio of mean length to upper half mean length), Rd (degree of reflectance, indicates how light or dark the fiber sample is), +b (yellowness, the measure of color pigmentation), leaf index (visual estimation of the amount of cotton plant leaf material is on the lint), and color grade (a function of Rd and +b of the fiber sample, based on the Nickerson-Hunter cotton colorimeter diagram) (Cotton Division, 1993). These fiber property values can then be used to calculate loan value (National Cotton Council, 2022) each year for the harvested cotton.

*Rotylenchulus reniformis* densities, which were taken in August or September (Ren), were analyzed using a LOG_10_(Ren+1) = LRen transformation. A mixed model analysis was performed on the year 1 and 2 treatments, since there was an unequal number of plots associated with each unique combination, using PROC GLIMMIX (SAS version 9.4, Cary, NC, U.S.A). The least square means were compared at *P* = 0.05, using the DIFF options to compare all pairwise least mean square differences. Since the 2022 data set had four replications for all nine treatments, that data set was analyzed using analysis of variance (PROC GLM, SAS version 9.4), and mean comparisons were made with the Waller-Duncan k-ratio t-test at *P* = 0.05. Similar analyses were done with lint yield in each of the three years. In year 1 there were three treatments (S1 [four plots], R1 [16 plots], and F1 [16 plots]). In year 2 there were six treatment combinations (S1S2 [4 plots], R1R2 [8 plots], F1F2 [4 plots], F1S2 [8 plots], F1R2 [4 plots], and R1S2 [8 plots]). In year 3 there were nine unique combinations (presented previously), each with four replications.

## Results

The initial plot soil samples taken in April of 2020, before planting, indicated that *R. reniformis* density averaged 926/100 cm^3^ soil and was not different between treatments. Rain from the previous year (September 2019 through April of 2020) was high (30.6 cm) for this area, and probably contributed to the excellent nematode recovery. Following year 1 (2020), the resistant cotton cultivar (DP 2143NR B3XF) yielded significantly higher (622 kg lint/ha) than the susceptible cotton cultivar (DP 2044 B3XF, 348 kg lint/ha). The loan values did not differ (*P* = 0.834) between the two cultivars. *Rotylenchulus reniformis* density in August was higher in the susceptible cotton plots (1,025 *R. reniformis*/100 cm^3^ soil, Lren = 2.95) than the resistant cotton (439 *R. reniformis*/100 cm^3^ soil, Lren = 2.63), and both cotton cultivars had significantly higher Lren counts than the fallow treatment (288 *R. reniformis*/100 cm^3^ soil, Lren = 2.40).

In year 2 (2021), lint yield was highest with two consecutive years of resistant cotton (916 kg lint/ha), and significantly higher than resistant cotton following fallow (815 kg lint/ha), or any of the year 2-susceptible cotton treatment combinations (Table 1). The lowest lint yield was associated with two years of susceptible cotton (516 kg lint/ha). The loan value did not differ between the two varieties (*P* = 0.864), or between any treatments (P = 0.847).

**Table 1.** Effect of cultivar and fallow rotation treatments in year 2 and year 3 on cotton lint yield and *Rotylenchulus reniformis* (REN) density.

**Treatment**	**Lint yield (kg/ha)**	**REN^b^**	**LREN**
2021			
R1R2	916 a	295	2.42 c
S1S2	516 d	835	2.89 a
F1F2	--------	60	1.73 d
R1S2	730 b	745	2.78 ab
F1R2	815 b	385	2.52 bc
F1S2	641 c	750	2.77 ab
2022			
R1R2R3	1,271 a	150	2.17 d
S1S2S3	597 d	505	2.64 ab
F1F2S3	823 c	295	2.33 cd
R1S2R3	1,051 b	265	2.36 bcd
R1S2S3	599 d	385	2.53 abc
F1R2S3	736 cd	655	2.78 a
F1S2F3	-------	150	2.12 d
F1S2S3	628 cd	570	2.75 a
R1R2S3	724 cd	395	2.54 abc

a R indicates the *R. reniformis* resistant cotton cultivar DP 2143NR B3XF was grown, S indicates the susceptible cotton cultivar DP 2044 B3XF was grown, and F indicates weed-free fallow. The crop in the first year is the first letter followed by 1, the second year is the second letter followed by 2, and third year is the third letter followed by 3.

b REN is *R. reniformis* soil population per 100 cm^3^ soil taken in September.

c LREN is the transformation of the *R. reniformis* density (LOG_10_(REN+1)).

*Rotylenchulus reniformis* densities in September of 2021 were highest for all the plots where susceptible cotton was grown in 2021, and lowest for plots with two years of fallow (Table 1). Transformed *R. reniformis* density was significantly higher in year 2 for the S1S2, R1S2, and F1S2 treatments compared with the R1R2, and F1F2 treatments. The F1R2 treatment was intermediate and not different from R1S2 and F1S2. It was clear from year 2, that regardless of history from year 1 (weed-free fallow, resistant or susceptible cotton cultivar), growing a susceptible cultivar resulted in substantial *R. reniformis* buildup by September. However, using a weed-free fallow system prior to growing a susceptible or resistant cotton cultivar did cause a yield penalty compared to using a resistant cultivar in year 1 (R1R2 yielded more than F1R2; and R1S2 yielded more than F1S2). The weed-free fallow in year 1 did not receive irrigation and there was only 30.5 cm of rain from May of 2020 through April of 2021.

In year 3 (2022), lint yield was higher in the three-year resistant cotton combination (R1R2R3) than all other combinations (Table 1). Lint yield was lower in all combinations that included a year 3 susceptible cotton cultivar than in the two combinations that had the resistant cotton cultivar in year 3 (R1R2R3 and R1S2R3). Loan value did not differ between the two varieties (*P* = 0.316) or between any treatments (*P* = 0.395).

*Rotylenchulus reniformis* transformed densities in September 2022 were significantly (*P* < 0.05) lower in both the resistant (2.27) and susceptible (2.59) cultivars than in 2020 (2.63 and 2.95, respectively) or 2021(2.45 and 2.80, respectively). This may be due to the extremely dry soil going into planting time (5.6 cm rain from September 2021 through April 2022), and the inability to maintain adequate soil moisture, even with irrigation during the hotter than normal summer months of 2022. The lowest Lren were associated with the R1R2R3, F1S2F3, F1F2S3, and R1S2R3. The highest Lren were associated with F1R2S3, F1S2S3, S1S2S3, R1S2S3, and R1R2S3. Those plots planted with susceptible cotton in year 3, in most cases, had the highest transformed *R. reniformis* densities. The exception was that after two years of fallow, the buildup on a susceptible cultivar was less than that found with most other year 3 susceptible cultivar cotton plots.

## Discussion

Currently, there are two companies that have *R. reniformis* resistant cultivars available, Phytogen (four commercially available cultivars as of 2022) and Deltapine (two commercially available cultivars as of 2022). The source of resistance in both company's cultivars are likely to be the same (*Gossypium barbadense* GB713 [Robinson et al., 2004]), though the only confirmation is in the PVP certificates available for PHY 332 W3FE and PHY 443 W3FE. The use of this excellent source of *R. reniformis* resistance (McCarty et al., 2013) was facilitated by the development of SSR markers associated with the resistance on chromosome 21 (Gutierrez et al., 2011). Cotton breeding lines with reniform nematode resistance that had been developed from crosses with GB713 yielded higher than susceptible commercial cotton varieties in *R. reniformis* infested field trials (Koebernick et al., 2021). The commercially available cultivars have not only obtained resistance to *R. reniformis*, but also to *M. incognita* (2-gene resistance), and in addition contain six transgenic traits (three Bt genes and three herbicide tolerant genes), making a total of at least nine genes that must be selected in addition to acceptable yield and fiber quality traits. Identification of markers for nematode resistance is essential for the development of commercial cultivars, particularly in a crop where transgenic traits are common. The two *M. incognita* resistance genes also have good SSR markers that can be utilized in commercial variety development (Gutiérrez et al., 2010; Jenkins et al., 2012).

The results in this study were as anticipated: fallow soil and the use of *R. reniformis* resistant cultivar DP 2143NR B3XF reduced *R. reniformis* density relative to a susceptible cultivar (DP 2044 B3XF). The yield advantage of using a resistant cultivar continuously was 78%, 77%, and 113% higher than the susceptible cultivar in year 1, 2, and 3, respectively. The advantage of using consecutive years of resistant cultivars was seen both in the reduction of *R. reniformis* density and improvement in lint yield. The economic value of using the reniform nematode resistant cultivar is close to the value of the yield difference, since the loan values for both cultivars were similar, and all other management costs would also have been similar. The cost of the seed in 2023 in this region was estimated at $1.47 per 1000 seed for DP 2044 B3XF and $1.87 per 1000 seed for DP 2143NR B3XF (Plains Cotton Growers Inc., 2023). For a planting density of 96,971 seed/ha, it would cost $38.79 more per hectare for the reniform nematode resistant variety than a susceptible variety, which is a minor expense considering the yield benefits.

One surprise in year 2 was that one year of fallow, followed by a resistant or susceptible cultivar, resulted in less yield than using a resistant cultivar in year 1, followed by a resistant or susceptible cultivar in year 2. That would suggest that something about the fallow treatment caused lower than expected yield in the following cotton crop. It is possible that there was higher than recovered *R. reniformis* populations in the fallow treatment, either because they were deeper in the soil profile than where sampling occurred, or because the existing nematode population was still immobilized after the 48-hour water extraction due to being in an anhydrobiotic state from dry soil (Womersley and Ching, 1989). The summer in 2020 was relatively dry (16 cm of rain from May – August). *Rotylenchulus reniformis* density in continuous cotton was found to be higher in the upper profile of field soil, compared to that of a corn-cotton rotation, where the nematode density peaked at the lower soil profile (Lee et al., 2015). It is also possible, though less likely, that the soil was drier going into the 2021 growing season in the rows that had been fallow in 2020, compared to the rows that had cotton in 2020 and were irrigated in-season. However, May 2021 was wet, which was why the plots were planted on 5 June in 2021, and so it is unlikely that initial differences in underlying moisture explained the yield differences in 2021.

The use of fallow ground rather than a nonhost rotation crop like corn or sorghum is only desirable under the scenario where water is limited and insufficient to grow an alternative crop to cotton. Corn or sorghum allow the outcome of farm income as well as the opportunity to reduce *R. reniformis* density (Davis et al., 2003; Stetina et al., 2007). However, in the semi-arid region where this work was conducted, producers already have insufficient irrigation capacity for their center pivot systems and may need to reduce the irrigated crop area to ¼ or ½ of the circle (Mitchell-McCallister et al., 2021). In this scenario, it would be more beneficial to use fallow as a tactic to reduce *R. reniformis* density rather than dryland cotton production, which could maintain nematode populations at damaging levels.

The results of this project support using continuous production of *R. reniformis* resistant cotton cultivars in infested fields. The profitability would be much higher than either using susceptible cultivars or using a weed-free fallow rotation with cotton. While the reduction in *R. reniformis* density was significant with the resistant cultivar, there can be sufficient population remaining to be damaging to susceptible cotton in the following year. It is likely that producers will want to repeatedly use the *R. reniformis* resistant cultivars. However, there is concern that repeated planting of a single source of resistance (presumably chromosome 21 Ren^barb1^ and/or Ren^barb2^ resistance QTL (Wubben et al. 2017; Gaudin and Wubber, 2021)) will ultimately result in the breakdown of that resistant source. The rotation of resistant and susceptible cultivars, or use of fallow or nonhosts of *R. reniformis* may delay that possibility.
